# Supplementary material for: Mapping the evolution of fertility support policies in China: A content and instrumental analysis
Source: PLoS One. 2025 Oct 9;20(10):e0332137. doi: 10.1371/journal.pone.0332137 (PMC12510515; doi:10.1371/journal.pone.0332137)
Supplement: S1 Appendix — (ZIP) [file pone.0332137.s001.zip › S1 Appendix. 226 original policy documents/72-国务院关于落实《政府工作报告》重点工作部门分工的意见(2019)(FBM-CLI-2-331114).doc]

国务院关于落实《政府工作报告》重点工作部门分工的意见(2019)

**发布部门：** [国务院](https://www.pkulaw.com/law/chl?Aggs.IssueDepartment=5)

**发文字号：**国发〔2019〕8号

**发布日期：**2019.03.29

**实施日期：**2019.03.29

**时效性：** [现行有效](https://www.pkulaw.com/law/chl?Aggs.TimelinessDic=01)

**效力级别：** [国务院规范性文件](https://www.pkulaw.com/law/chl?Aggs.EffectivenessDic=XC0203)

**法规类别：** [行政机关](https://www.pkulaw.com/law/chl?Aggs.Category=00202) [营商环境优化](https://dobusiness.pkulaw.com/)

国务院关于落实《政府工作报告》重点工作部门分工的意见
（国发〔2019〕8号）

各省、自治区、直辖市人民政府，国务院各部委、各直属机构：
　　为全面贯彻党的十九大和十九届二中、三中全会精神，深入落实中央经济工作会议精神和十三届全国人大二次会议通过的《政府工作报告》部署，做好今年政府各项工作，实现经济社会发展目标任务，现就《政府工作报告》确定的重点工作，提出部门分工意见如下：
　　**一、**落实2019年经济社会发展总体要求和政策取向
　　（一）总体要求和主要预期目标。
　　1.在以习近平同志为核心的党中央坚强领导下，以习近平新时代中国特色社会主义思想为指导，全面贯彻党的十九大和十九届二中、三中全会精神，统筹推进“五位一体”总体布局，协调推进“四个全面”战略布局，坚持稳中求进工作总基调，坚持新发展理念，坚持推动高质量发展，坚持以供给侧结构性改革为主线，坚持深化市场化改革、扩大高水平开放，加快建设现代化经济体系，继续打好三大攻坚战，着力激发微观主体活力，创新和完善宏观调控，统筹推进稳增长、促改革、调结构、惠民生、防风险、保稳定工作，保持经济运行在合理区间，进一步稳就业、稳金融、稳外贸、稳外资、稳投资、稳预期，提振市场信心，增强人民群众获得感、幸福感、安全感，保持经济持续健康发展和社会大局稳定。（国务院各部门分别负责）
　　2.国内生产总值增长6%-6.5%；城镇新增就业1100万人以上，城镇调查失业率5.5%左右，城镇登记失业率4.5%以内；居民消费价格涨幅3%左右；国际收支基本平衡，进出口稳中提质；宏观杠杆率基本稳定，金融财政风险有效防控；农村贫困人口减少1000万以上，居民收入增长与经济增长基本同步；生态环境进一步改善，单位国内生产总值能耗下降3%左右，主要污染物排放量继续下降。（国家发展改革委、财政部、人力资源社会保障部、生态环境部、商务部、人民银行、海关总署、银保监会、国家外汇局、国务院扶贫办等按职责分工负责）
　　（二）积极的财政政策要加力提效。
　　3.赤字率拟按2.8%安排，比去年预算高0.2个百分点；财政赤字2.76万亿元，其中中央财政赤字1.83万亿元，地方财政赤字9300亿元。今年财政支出超过23万亿元，增长6.5%。中央对地方均衡性转移支付增长10.9%。改革完善县级基本财力保障机制，缓解困难地区财政运转压力，决不让基本民生保障出问题。（财政部牵头）
　　（三）稳健的货币政策要松紧适度。
　　4.广义货币M2和社会融资规模增速要与国内生产总值名义增速相匹配。灵活运用多种货币政策工具，疏通货币政策传导渠道，保持流动性合理充裕。深化利率市场化改革，降低实际利率水平。完善汇率形成机制，保持人民币汇率在合理均衡水平上的基本稳定。（人民银行、银保监会、国家外汇局等按职责分工负责）
　　（四）就业优先政策要全面发力。
　　5.城镇新增就业要在实现预期目标的基础上，力争达到近几年的实际规模，既保障城镇劳动力就业，也为农业富余劳动力转移就业留出空间。（人力资源社会保障部、国家发展改革委、财政部、人民银行等按职责分工负责）
　　（五）继续坚持以供给侧结构性改革为主线。
　　6.巩固“三去一降一补”成果，增强微观主体活力，提升产业链水平，畅通国民经济循环，推动经济高质量发展。（国家发展改革委、工业和信息化部、财政部、住房城乡建设部、人民银行、国务院国资委等按职责分工负责）
　　（六）继续打好三大攻坚战。
　　7.稳妥处理地方政府债务风险。（财政部牵头）防范金融市场异常波动。（人民银行牵头）防控输入性风险。（国家发展改革委、财政部、国务院金融稳定发展委员会办公室、人民银行、银保监会、证监会、国家外汇局等按职责分工负责）
　　8.精准脱贫要坚持现行标准，聚焦深度贫困地区和特殊贫困群体，加大攻坚力度，提高脱贫质量。（国务院扶贫办牵头）
　　9.污染防治要聚焦打赢蓝天保卫战等重点任务，统筹兼顾、标本兼治，使生态环境质量持续改善。（生态环境部牵头）

　　**二、**继续创新和完善宏观调控，确保经济运行在合理区间
　　（七）实施更大规模的减税。
　　10.普惠性减税与结构性减税并举，重点降低制造业和小微企业税收负担。深化增值税改革，将制造业等行业现行16%的税率降至13%，将交通运输业、建筑业等行业现行10%的税率降至9%，确保主要行业税负明显降低；保持6%一档的税率不变，但通过采取对生产、生活性服务业增加税收抵扣等配套措施，确保所有行业税负只减不增，继续向推进税率三档并两档、税制简化方向迈进。抓好年初出台的小微企业普惠性减税政策落实。（财政部、税务总局牵头）
　　（八）明显降低企业社保缴费负担。
　　11.下调城镇职工基本养老保险单位缴费比例，各地可降至16%。继续执行阶段性降低失业和工伤保险费率政策。（人力资源社会保障部、财政部牵头）稳定现行征缴方式，各地在征收体制改革过程中不得采取增加小微企业实际缴费负担的做法，不得自行对历史欠费进行集中清缴。（人力资源社会保障部、税务总局牵头）加快推进养老保险省级统筹改革。继续提高企业职工基本养老保险基金中央调剂比例。（人力资源社会保障部、财政部牵头）划转部分国有资本充实社保基金。（财政部牵头）
　　（九）确保减税降费落实到位。
　　12.全年减轻企业税收和社保缴费负担近2万亿元。（财政部、国家发展改革委、人力资源社会保障部、税务总局等按职责分工负责）中央财政要开源节流，增加特定国有金融机构和央企上缴利润，一般性支出压减5%以上、“三公”经费再压减3%左右，长期沉淀资金一律收回。地方政府要大力优化支出结构，多渠道盘活各类资金和资产。（财政部牵头）
　　（十）着力缓解企业融资难融资贵问题。
　　13.适时运用存款准备金率、利率等数量和价格手段，引导金融机构扩大信贷投放、降低贷款成本，精准有效支持实体经济。加大对中小银行定向降准力度，释放的资金全部用于民营和小微企业贷款。支持大型商业银行多渠道补充资本，增强信贷投放能力，鼓励增加制造业中长期贷款和信用贷款。今年国有大型商业银行小微企业贷款要增长30%以上。完善金融机构内部考核机制，激励加强普惠金融服务，切实使中小微企业融资紧张状况有明显改善。今年小微企业融资成本在2018年基础上再降低1个百分点。（人民银行、银保监会、证监会、财政部等按职责分工负责）
　　14.清理规范银行及中介服务收费。（银保监会、国家发展改革委、市场监管总局等按职责分工负责）
　　（十一）有效发挥地方政府债券作用。
　　15.拟安排地方政府专项债券2.15万亿元，比去年增加8000亿元，为重点项目建设提供资金支持，也为更好防范化解地方政府债务风险创造条件。合理扩大专项债券使用范围。继续发行一定数量的地方政府置换债券，减轻地方利息负担。（财政部牵头）
　　16.鼓励采取市场化方式，妥善解决融资平台到期债务问题。（财政部、国家发展改革委、人民银行、银保监会等按职责分工负责）
　　（十二）多管齐下稳定和扩大就业。
　　17.扎实做好高校毕业生、退役军人、农民工等重点群体就业工作，加强对城镇各类就业困难人员的就业帮扶。（人力资源社会保障部、教育部、退役军人部等按职责分工负责）对招用农村贫困人口、城镇登记失业半年以上人员的各类企业，三年内给予定额税费减免。（财政部、税务总局牵头）
　　18.加强对灵活就业、新就业形态的支持。实施职业技能提升行动，从失业保险基金结余中拿出1000亿元，用于1500万人次以上的职工技能提升和转岗转业培训。健全技术工人职业发展机制和政策。（人力资源社会保障部牵头）
　　19.改革完善高职院校考试招生办法，鼓励更多应届高中毕业生和退役军人、下岗职工、农民工等报考，今年大规模扩招100万人。（教育部牵头）加快学历证书和职业技能等级证书互通衔接。改革高职院校办学体制，加强师资队伍建设，提高办学质量。引导一批普通本科高校转为应用型大学。（教育部、人力资源社会保障部牵头）扩大高职院校奖助学金覆盖面、提高补助标准。中央财政大幅增加对高职院校的投入，地方财政也要加强支持。设立中等职业教育国家奖学金。（财政部、教育部牵头）支持企业和社会力量兴办职业教育，加快产教融合实训基地建设。加快发展现代职业教育，加快培养国家发展急需的各类技术技能人才。（教育部、国家发展改革委、人力资源社会保障部、国务院国资委等按职责分工负责）

　　**三、**激发市场主体活力，着力优化营商环境
　　（十三）以简审批优服务便利投资兴业。
　　20.深化“放管服”改革，降低制度性交易成本。政府要坚决把不该管的事项交给市场，最大限度减少对资源的直接配置，审批事项应减尽减，确需审批的要简化流程和环节。推行网上审批和服务，抓紧建成全国一体化在线政务服务平台，加快实现一网通办、异地可办，使更多事项不见面办理，确需到现场办的要“一窗受理、限时办结”“最多跑一次”。建立政务服务“好差评”制度，服务绩效由企业和群众来评判。（国务院办公厅牵头，有关部门按职责分工负责）
　　21.进一步缩减[市场准入负面清单](https://www.pkulaw.com/chl/d8135b05437494cebdfb.html)，推动“非禁即入”普遍落实。（国家发展改革委、商务部牵头）今年要对所有涉企经营许可事项实行“证照分离”改革，使企业更便捷拿到营业执照并尽快正常运营，坚决克服“准入不准营”的现象。（国务院办公厅、市场监管总局、司法部等按职责分工负责）今年要力争在全国将企业开办时间压缩至5个工作日以内，鼓励有条件的地方进一步压缩至3个工作日以内。（市场监管总局牵头）今年要在全国推开工程建设项目审批制度改革，使全流程审批时间大幅缩短。（住房城乡建设部牵头）要继续压缩专利审查和商标注册时间。（国家知识产权局牵头）持续开展“减证便民”改革行动，不能让繁琐证明来回折腾企业和群众。（司法部牵头）
　　（十四）以公正监管促进公平竞争。
　　22.改革完善公平竞争审查和公正监管制度，加快清理妨碍统一市场和公平竞争的各种规定和做法。推进“双随机、一公开”跨部门联合监管。（市场监管总局牵头）推行信用监管改革。完善失信联合惩戒机制，促进各类市场主体守法诚信经营。（国家发展改革委、人民银行、市场监管总局等按职责分工负责）推行“互联网＋监管”改革。（国务院办公厅牵头，有关部门按职责分工负责）
　　23.优化环保、消防、税务、市场监管等执法方式。深化综合行政执法改革，清理规范行政处罚事项，坚决治理多头检查、重复检查。对监管者也要强监管、立规矩，决不允许搞选择性执法、任性执法，决不允许刁难企业和群众。（生态环境部、住房城乡建设部、交通运输部、农业农村部、文化和旅游部、应急部、税务总局、市场监管总局、司法部等按职责分工负责）依法打击制售假冒伪劣商品等违法行为，让严重违法者付出付不起的代价。（市场监管总局牵头）
　　（十五）以改革推动降低涉企收费。
　　24.深化电力市场化改革，清理电价附加收费，降低制造业用电成本，一般工商业平均电价再降低10%。（国家发展改革委、财政部、国家能源局等按职责分工负责）
　　25.深化收费公路制度改革，推动降低过路过桥费用，治理对客货运车辆不合理审批和乱收费、乱罚款。两年内基本取消全国高速公路省界收费站，实现不停车快捷收费，减少拥堵、便利群众。（交通运输部牵头）取消或降低一批铁路、港口收费。（国家发展改革委、交通运输部、中国铁路总公司等按职责分工负责）
　　26.专项治理中介服务收费。继续清理规范行政事业性收费。加快收费清单“一张网”建设。（国家发展改革委、财政部、民政部、市场监管总局等按职责分工负责）

　　**四、**坚持创新引领发展，培育壮大新动能
　　（十六）推动传统产业改造提升。
　　27.围绕推动制造业高质量发展，强化工业基础和技术创新能力，促进先进制造业和现代服务业融合发展，加快建设制造强国。打造工业互联网平台，拓展“智能＋”，为制造业转型升级赋能。（工业和信息化部、国家发展改革委、科技部等按职责分工负责）
　　28.支持企业加快技术改造和设备更新，将固定资产加速折旧优惠政策扩大至全部制造业领域。（工业和信息化部、国家发展改革委、财政部、税务总局等按职责分工负责）强化质量基础支撑，推动标准与国际先进水平对接，提升产品和服务品质。（市场监管总局、工业和信息化部等按职责分工负责）
　　（十七）促进新兴产业加快发展。
　　29.深化大数据、人工智能等研发应用，培育新一代信息技术、高端装备、生物医药、新能源汽车、新材料等新兴产业集群，壮大数字经济。（国家发展改革委、工业和信息化部、科技部等按职责分工负责）坚持包容审慎监管，支持新业态新模式发展，促进平台经济、共享经济健康成长。加快在各行业各领域推进“互联网＋”。（国家发展改革委、工业和信息化部、交通运输部、商务部、市场监管总局等按职责分工负责）
　　30.持续推动网络提速降费。开展城市千兆宽带入户示范，改造提升远程教育、远程医疗网络，推动移动网络扩容升级。今年中小企业宽带平均资费再降低15%，移动网络流量平均资费再降低20%以上，在全国实行“携号转网”，规范套餐设置，使降费实实在在、消费者明明白白。（工业和信息化部牵头）
　　（十八）提升科技支撑能力。
　　31.加大基础研究和应用基础研究支持力度，强化原始创新，加强关键核心技术攻关。抓紧布局国家实验室，重组国家重点实验室体系。完善重大科技项目组织管理。健全以企业为主体的产学研一体化创新机制，支持企业牵头实施重大科技项目。（科技部、国家发展改革委、工业和信息化部、教育部、财政部、国务院国资委、国家国防科工局等按职责分工负责）加快建设科技创新资源开放共享平台，强化对中小企业的技术创新服务。扩大国际创新合作。（科技部牵头）
　　32.全面加强知识产权保护，健全知识产权侵权惩罚性赔偿制度，促进发明创造和转化运用。（国家知识产权局、市场监管总局等按职责分工负责）
　　33.赋予创新团队和领军人才更大的人财物支配权和技术路线决策权。进一步提高基础研究项目间接经费占比，开展项目经费使用“包干制”改革试点，不设科目比例限制，由科研团队自主决定使用。（科技部、财政部、教育部等按职责分工负责）完善科技成果评价机制。（科技部牵头）加强科研伦理和学风建设。（科技部、中国科协牵头）
　　（十九）进一步把大众创业万众创新引向深入。
　　34.鼓励更多社会主体创新创业，加强全方位服务，发挥双创示范基地带动作用。（国家发展改革委牵头）强化普惠性支持，落实好小规模纳税人增值税起征点从月销售额3万元提高到10万元等税收优惠政策。（财政部、税务总局牵头）改革完善人才培养、使用、评价机制，优化归国留学人员和外籍人才服务。（人力资源社会保障部、科技部、教育部、国家移民局等按职责分工负责）
　　35.设立科创板并试点注册制。（证监会牵头）鼓励发行双创金融债券，扩大知识产权质押融资。（人民银行、银保监会、证监会、国家知识产权局等按职责分工负责）支持发展创业投资。（国家发展改革委、证监会、财政部等按职责分工负责）

　　**五、**促进形成强大国内市场，持续释放内需潜力
　　（二十）推动消费稳定增长。
　　36.多措并举促进城乡居民增收，增强消费能力。落实好新修订的[个人所得税法](https://www.pkulaw.com/chl/d74fa423af05cffdbdfb.html)，使符合减税政策的约8000万纳税人应享尽享。（国家发展改革委、财政部、税务总局等按职责分工负责）发展消费新业态新模式，促进线上线下消费融合发展，培育消费新增长点。（国家发展改革委、商务部等按职责分工负责）
　　37.大力发展养老特别是社区养老服务业，对在社区提供日间照料、康复护理、助餐助行等服务的机构给予税费减免、资金支持、水电气热价格优惠等扶持。（民政部、国家发展改革委、财政部、税务总局等按职责分工负责）新建居住区应配套建设社区养老服务设施，加强农村养老服务设施建设。实施“互联网＋养老”行动。（民政部、国家发展改革委、住房城乡建设部等按职责分工负责）
　　38.改革完善医养结合政策。（国家卫生健康委、民政部等按职责分工负责）扩大长期护理保险制度试点。（国家医保局牵头）要针对实施全面两孩政策后的新情况，加快发展社区托幼服务等多种形式的婴幼儿照护服务，支持社会力量兴办托育服务机构。（国家卫生健康委、教育部、国家发展改革委、全国妇联等按职责分工负责）加强儿童安全保障。（教育部、公安部、国家卫生健康委等按职责分工负责）促进家政服务业提质扩容。（国家发展改革委、商务部等按职责分工负责）
　　39.发展全域旅游，壮大旅游产业。（文化和旅游部、国家发展改革委等按职责分工负责）稳定汽车消费，继续执行新能源汽车购置优惠政策，推动充电、加氢等设施建设。（财政部、工业和信息化部、国家发展改革委、商务部、交通运输部、住房城乡建设部、国家能源局等按职责分工负责）健全农村流通网络，支持电商发展。（商务部牵头）支持快递发展。（国家邮政局牵头）
　　（二十一）合理扩大有效投资。
　　40.紧扣国家发展战略，加快实施一批重点项目。完成铁路投资8000亿元、公路水运投资1.8万亿元，再开工一批重大水利工程，加快川藏铁路规划建设，加大城际交通、物流、市政、灾害防治、民用和通用航空等基础设施投资力度，加强新一代信息基础设施建设。（国家发展改革委、工业和信息化部、财政部、住房城乡建设部、交通运输部、水利部、应急部、中国铁路总公司、中国民航局、开发银行、进出口银行等按职责分工负责）
　　41.今年中央预算内投资安排5776亿元，比去年增加400亿元。创新项目融资方式，适当降低基础设施等项目资本金比例。（国家发展改革委牵头）用好开发性金融工具，吸引更多民间资本参与重点领域项目建设。落实民间投资支持政策，有序推进政府和社会资本合作。（国家发展改革委、财政部、人民银行、银保监会、开发银行等按职责分工负责）改革完善招投标制度。（国家发展改革委牵头）
　　42.对拖欠企业的款项年底前要清偿一半以上，决不允许增加新的拖欠。（工业和信息化部、财政部、国务院国资委牵头）

　　**六、**对标全面建成小康社会任务，扎实推进脱贫攻坚和乡村振兴
　　（二十二）打好精准脱贫攻坚战。
　　43.重点解决实现“两不愁三保障”面临的突出问题，加大“三区三州”等深度贫困地区脱贫攻坚力度，加强基础设施建设，落实对特殊贫困人口的保障措施。（国务院扶贫办牵头）要大力扶持贫困地区发展特色优势产业。（农业农村部牵头）
　　44.开展贫困地区控辍保学专项行动，明显降低辍学率。继续增加重点高校专项招收农村和贫困地区学生人数。（教育部牵头）
　　45.基本完成“十三五”易地扶贫搬迁规划建设任务，加强后续扶持。（国家发展改革委牵头）对摘帽县和脱贫人口的扶持政策要保持一段时间，巩固脱贫成果。完善考核监督，用好中央脱贫攻坚专项巡视成果。（国务院扶贫办牵头）
　　（二十三）抓好农业特别是粮食生产。
　　46.稳定粮食产量，优化品种结构。加强农田水利建设，新增高标准农田8000万亩以上。稳定生猪等畜禽生产，做好非洲猪瘟等疫病防控。加快农业科技改革创新，大力发展现代种业，加强先进实用技术推广，推进农业全程机械化。培育家庭农场、农民合作社等新型经营主体，加强面向小农户的社会化服务，发展多种形式规模经营。（农业农村部牵头）扶持主产区发展农产品精深加工。支持返乡入乡创业创新，推动一二三产业融合发展，壮大县域经济。（农业农村部、国家发展改革委等按职责分工负责）实施地理标志农产品保护工程。（农业农村部、国家知识产权局等按职责分工负责）
　　47.根治拖欠农民工工资问题，抓紧制定专门行政法规，确保农民工按时拿到应有的报酬。（人力资源社会保障部、司法部等按职责分工负责）
　　（二十四）扎实推进乡村建设。
　　48.科学编制和实施建设规划，大力改善生产生活条件。（自然资源部、农业农村部、住房城乡建设部等按职责分工负责）加快实施农村饮水安全巩固提升工程，今明两年要解决好饮水困难人口的饮水安全问题，提高6000万农村人口供水保障水平。（水利部牵头）完成新一轮农村电网升级改造。（国家发展改革委、国家能源局牵头）新建改建农村公路20万公里。（交通运输部牵头）继续推进农村危房改造。（住房城乡建设部、国家发展改革委、财政部等按职责分工负责）因地制宜开展农村人居环境整治，推进“厕所革命”、垃圾污水治理，建设美丽乡村。（农业农村部、生态环境部、住房城乡建设部、财政部、国家发展改革委等按职责分工负责）
　　（二十五）全面深化农村改革。
　　49.推广农村土地征收、集体经营性建设用地入市、宅基地制度改革试点成果。深化集体产权、集体林权、国有林区林场、农垦、供销社等改革。（自然资源部、农业农村部、国家林草局、供销合作总社等按职责分工负责）
　　50.改革完善农业支持保护体系，健全粮食价格市场化形成机制，扩大政策性农业保险改革试点，创新和加强农村金融服务。（农业农村部、国家发展改革委、财政部、商务部、人民银行、银保监会、农业发展银行等按职责分工负责）

　　**七、**促进区域协调发展，提高新型城镇化质量
　　（二十六）优化区域发展格局。
　　51.制定西部开发开放新的政策措施，西部地区企业所得税优惠等政策到期后继续执行。（国家发展改革委、财政部、税务总局等按职责分工负责）落实和完善促进东北全面振兴、中部地区崛起、东部率先发展的改革创新举措。（国家发展改革委牵头）
　　52.京津冀协同发展重在疏解北京非首都功能，高标准建设雄安新区。落实粤港澳大湾区发展规划纲要，促进规则衔接，推动生产要素流动和人员往来便利化。将长三角区域一体化发展上升为国家战略，编制实施发展规划纲要。长江经济带发展要坚持上中下游协同，加强生态保护修复和综合交通运输体系建设，打造高质量发展经济带。（国家发展改革委牵头）
　　53.支持资源型地区经济转型。加快补齐革命老区、民族地区、边疆地区、贫困地区发展短板。（国家发展改革委牵头）大力发展蓝色经济，保护海洋环境，建设海洋强国。（国家发展改革委、自然资源部牵头）
　　（二十七）深入推进新型城镇化。
　　54.抓好农业转移人口落户，推动城镇基本公共服务覆盖常住人口。（公安部、国家发展改革委等按职责分工负责）
　　55.更好解决群众住房问题，落实城市主体责任，改革完善住房市场体系和保障体系，促进房地产市场平稳健康发展。继续推进保障性住房建设和城镇棚户区改造，保障困难群体基本居住需求。继续推进地下综合管廊建设。城镇老旧小区量大面广，要大力进行改造提升，更新水电路气等配套设施，支持加装电梯和无障碍环境建设。（住房城乡建设部牵头）健全便民市场、便利店、步行街、停车场等生活服务设施。（住房城乡建设部、商务部等按职责分工负责）

　　**八、**加强污染防治和生态建设，大力推动绿色发展
　　（二十八）持续推进污染防治。
　　56.巩固扩大蓝天保卫战成果，今年二氧化硫、氮氧化物排放量要下降3%，重点地区细颗粒物（PM2.5）浓度继续下降。持续开展京津冀及周边、长三角、汾渭平原大气污染治理攻坚，加强工业、燃煤、机动车三大污染源治理。（生态环境部牵头）做好北方地区清洁取暖工作，确保群众温暖过冬。（国家能源局、国家发展改革委、生态环境部等按职责分工负责）
　　57.强化水、土壤污染防治，今年化学需氧量、氨氮排放量要下降2%。（生态环境部牵头）加快治理黑臭水体，防治农业面源污染，推进重点流域和近岸海域综合整治。加强固体废弃物和城市垃圾分类处置，促进减量化、资源化、无害化。加强污染防治重大科技攻关。（生态环境部、住房城乡建设部、科技部、农业农村部、国家卫生健康委等按职责分工负责）
　　58.改革创新环境治理方式，对企业既依法依规监管，又重视合理诉求、加强帮扶指导，对需要达标整改的给予合理过渡期，避免处置措施简单粗暴、一关了之。（生态环境部牵头）
　　（二十九）壮大绿色环保产业。
　　59.坚持源头治理，加快火电、钢铁行业超低排放改造，实施重污染行业达标排放改造。（国家能源局、生态环境部牵头）调整优化能源结构。推进煤炭清洁化利用。健全天然气产供储销体系。大力发展可再生能源，加快解决风、光、水电消纳问题。（国家发展改革委、国家能源局牵头）促进资源节约集约和循环利用。（国家发展改革委牵头）
　　60.加大城市污水管网和处理设施建设力度。（住房城乡建设部、生态环境部、国家发展改革委、财政部等按职责分工负责）推广绿色建筑、绿色快递包装。（住房城乡建设部、交通运输部、国家邮政局等按职责分工负责）改革完善环境经济政策，健全排污权交易制度。（国家发展改革委、生态环境部、财政部等按职责分工负责）加快发展绿色金融，培育一批专业化环保骨干企业，提升绿色发展能力。（国家发展改革委、生态环境部、人民银行等按职责分工负责）
　　（三十）加强生态系统保护修复。
　　61.推进山水林田湖草生态保护修复工程试点。（财政部、自然资源部、生态环境部等按职责分工负责）持续抓好国土绿化，加强荒漠化、石漠化、水土流失治理。加大生物多样性保护力度。继续开展退耕还林还草还湿。深化国家公园体制改革。（自然资源部、生态环境部、水利部、国家林草局、国家发展改革委等按职责分工负责）健全生态补偿机制。（国家发展改革委、财政部、自然资源部、生态环境部等按职责分工负责）

　　**九、**深化重点领域改革，加快完善市场机制
　　（三十一）加快国资国企改革。
　　62.加强和完善国有资产监管，推进国有资本投资、运营公司改革试点，促进国有资产保值增值。（国务院国资委、财政部等按职责分工负责）积极稳妥推进混合所有制改革。（国家发展改革委、国务院国资委牵头）完善公司治理结构，健全市场化经营机制，建立职业经理人等制度。（国务院国资委牵头）
　　63.依法处置“僵尸企业”。（国家发展改革委、国务院国资委牵头）深化电力、油气、铁路等领域改革，自然垄断行业要根据不同行业特点实行网运分开，将竞争性业务全面推向市场。（国家发展改革委、国务院国资委牵头，财政部、交通运输部等按职责分工负责）
　　（三十二）下大气力优化民营经济发展环境。
　　64.坚持“两个毫不动摇”，鼓励、支持、引导非公有制经济发展。（国家发展改革委、工业和信息化部、全国工商联等按职责分工负责）按照竞争中性原则，在要素获取、准入许可、经营运行、政府采购和招投标等方面，对各类所有制企业平等对待。（国家发展改革委、工业和信息化部、财政部等按职责分工负责）
　　65.构建亲清新型政商关系，健全政企沟通机制，激发企业家精神，促进民营经济发展升级。（国家发展改革委、工业和信息化部、财政部、全国工商联等按职责分工负责）保护产权必须坚定不移，对侵权行为要依法惩处，对错案冤案要有错必纠。（国家发展改革委、公安部、司法部、市场监管总局、国家知识产权局等按职责分工负责）
　　（三十三）深化财税金融体制改革。
　　66.加大预算公开改革力度，全面实施预算绩效管理。深化中央与地方财政事权和支出责任划分改革，推进中央与地方收入划分改革。完善转移支付制度。规范地方政府举债融资机制。（财政部牵头）健全地方税体系。（财政部、税务总局牵头）稳步推进房地产税立法。（有关部门按职责分工配合做好工作）
　　67.以服务实体经济为导向，改革优化金融体系结构，发展民营银行和社区银行。（银保监会、人民银行牵头）增强保险业风险保障功能。（银保监会牵头）改革完善资本市场基础制度，促进多层次资本市场健康稳定发展，提高直接融资特别是股权融资比重。（证监会牵头）

　　**十、**推动全方位对外开放，培育国际经济合作和竞争新优势
　　（三十四）促进外贸稳中提质。
　　68.推动出口市场多元化。扩大出口信用保险覆盖面。改革完善跨境电商等新业态扶持政策。推动服务贸易创新发展，引导加工贸易转型升级、向中西部转移。优化进口结构，积极扩大进口。办好第二届中国国际进口博览会。（商务部牵头）发挥好综合保税区作用。加快提升通关便利化水平。（海关总署牵头）
　　（三十五）加大吸引外资力度。
　　69.进一步放宽市场准入，缩减外资准入负面清单，允许更多领域实行外资独资经营。（国家发展改革委会同商务部负责）加快与国际通行经贸规则对接，提高政策透明度和执行一致性，营造内外资企业一视同仁、公平竞争的公正市场环境。加强外商合法权益保护。（国家发展改革委、商务部等按职责分工负责）
　　70.落实金融等行业改革开放举措，完善债券市场开放政策。（人民银行、银保监会、证监会、国家外汇局、国家发展改革委、商务部等按职责分工负责）
　　71.赋予自贸试验区更大改革创新自主权，增设上海自贸试验区新片区。（商务部牵头）推进海南自贸试验区建设、探索建设中国特色自由贸易港。（商务部牵头，国家发展改革委等按职责分工负责）支持国家级经开区、高新区、新区开展自贸试验区相关改革试点，增强辐射带动作用，打造改革开放新高地。（商务部、国家发展改革委、科技部等按职责分工负责）
　　（三十六）推动共建“一带一路”。
　　72.坚持共商共建共享，遵循市场原则和国际通行规则，发挥企业主体作用，推动基础设施互联互通，加强国际产能合作，拓展第三方市场合作。推动对外投资合作健康有序发展。（国家发展改革委、商务部等按职责分工负责）办好第二届“一带一路”国际合作高峰论坛。（外交部、国家发展改革委、商务部等按职责分工负责）
　　（三十七）促进贸易和投资自由化便利化。
　　73.坚定维护经济全球化和自由贸易，积极参与世贸组织改革。加快构建高标准自贸区网络，推进区域全面经济伙伴关系协定、中日韩自贸区、中欧投资协定谈判，继续推动中美经贸磋商。对作出的承诺认真履行，对自身合法权益坚决维护。（商务部牵头）

　　**十一、**加快发展社会事业，更好保障和改善民生
　　（三十八）发展更加公平更有质量的教育。
　　74.深化教育教学改革。推进城乡义务教育一体化发展，加快改善乡村学校办学条件，加强乡村教师队伍建设，抓紧解决城镇学校“大班额”问题，保障进城务工人员随迁子女教育，发展“互联网＋教育”，促进优质资源共享。（教育部牵头）
　　75.多渠道扩大学前教育供给，无论是公办还是民办幼儿园，只要符合安全标准、收费合理、家长放心，政府都要支持。（教育部、国家发展改革委、住房城乡建设部等按职责分工负责）推进高中阶段教育普及，办好民族教育、特殊教育、继续教育，依法支持民办教育发展。（教育部牵头）持续抓好义务教育教师工资待遇落实。（人力资源社会保障部、教育部、财政部等按职责分工负责）推进一流大学和一流学科建设，支持中西部建设有特色、高水平大学。（教育部、国家发展改革委、财政部等按职责分工负责）
　　76.国家财政性教育经费占国内生产总值比例继续保持在4%以上，中央财政教育支出安排超过1万亿元。（财政部、教育部牵头）
　　（三十九）保障基本医疗卫生服务。
　　77.继续提高城乡居民基本医保和大病保险保障水平，居民医保人均财政补助标准增加30元，一半用于大病保险。（财政部、国家医保局牵头）降低并统一大病保险起付线，报销比例由50%提高到60%。深化医保支付方式改革，优化医保支出结构。抓紧落实和完善跨省异地就医直接结算政策，尽快使异地就医患者在所有定点医院能持卡看病、即时结算。（国家医保局牵头）完善药品集中采购和使用机制。（国家医保局、国家卫生健康委、国家药监局等按职责分工负责）
　　78.加强重大疾病防治。要实施癌症防治行动，推进预防筛查、早诊早治和科研攻关。加快儿童药物研发。加强罕见病用药保障。（国家卫生健康委、科技部、国家医保局、国家药监局等按职责分工负责）抓好传染病、地方病防治。做好常见慢性病防治。（国家卫生健康委牵头）抓好青少年近视防治。（教育部、国家卫生健康委牵头）把高血压、糖尿病等门诊用药纳入医保报销。（国家医保局牵头）
　　79.深化公立医院综合改革。促进社会办医。发展“互联网＋医疗健康”，加快建立远程医疗服务体系，加强基层医疗卫生机构能力建设和医护人员培养，提升分级诊疗和家庭医生签约服务质量。加强健康教育和健康管理。坚持预防为主，将新增基本公共卫生服务财政补助经费全部用于村和社区。（国家卫生健康委、财政部、国家发展改革委、教育部等按职责分工负责）
　　80.完善生育配套政策，加强妇幼保健服务。（国家卫生健康委牵头）支持中医药事业传承创新发展。（国家卫生健康委、国家中医药局等按职责分工负责）药品疫苗攸关生命安全，必须强化全程监管，对违法者要严惩不贷，对失职渎职者要严肃查办。（国家药监局、国家卫生健康委等按职责分工负责）
　　（四十）完善社会保障制度和政策。
　　81.推进多层次养老保障体系建设。（人力资源社会保障部、财政部等按职责分工负责）继续提高退休人员基本养老金。（人力资源社会保障部、财政部牵头）落实退役军人待遇保障，完善退役士兵基本养老、基本医疗保险接续政策。（退役军人部、人力资源社会保障部、国家医保局等按职责分工负责）适当提高城乡低保、专项救助等标准，加强困境儿童保障。加大城镇困难职工脱困力度。提升残疾预防和康复服务水平。（民政部、财政部、国务院妇儿工委办公室、全国总工会、中国残联等按职责分工负责）
　　（四十一）丰富人民群众精神文化生活。
　　82.加强文物保护利用和非物质文化遗产传承。推动文化事业和文化产业改革发展，提升基层公共文化服务能力。（文化和旅游部、国家文物局、国家发展改革委等按职责分工负责）扎实做好2020年奥运会、残奥会备战工作。（体育总局、中国残联等按职责分工负责）精心筹办北京冬奥会、冬残奥会。办好第七届世界军人运动会。（有关部门按职责分工负责）
　　（四十二）加强和创新社会治理。
　　83.推动社会治理重心向基层下移，推广促进社会和谐的“枫桥经验”，构建城乡社区治理新格局。引导支持社会组织、人道救助、志愿服务和慈善事业健康发展。保障妇女、儿童、老人、残疾人合法权益。（民政部、公安部、国务院妇儿工委办公室、全国老龄办、全国妇联、中国残联、中国红十字会总会等按职责分工负责）健全社会信用体系。（国家发展改革委、人民银行牵头）
　　84.改进信访工作，化解信访突出矛盾，依法及时解决群众合理诉求。（国家信访局牵头，有关部门按职责分工负责）
　　85.健全国家应急体系，提高防灾减灾救灾能力。加强安全生产，防范遏制重特大事故。做好地震、气象、水文、地质、测绘等工作。（应急部牵头，有关部门按职责分工负责）
　　86.加强国家安全能力建设。完善立体化社会治安防控体系，深入推进扫黑除恶专项斗争，依法惩治盗抢骗黄赌毒等违法犯罪活动，打击非法集资、传销等经济犯罪，整治侵犯公民个人信息等突出问题。（公安部、安全部、人民银行、银保监会等按职责分工负责）

　　**十二、**全面加强政府自身建设
　　（四十三）坚持依法全面履职。
　　87.深入贯彻全面依法治国基本方略，严格遵守[宪法](https://www.pkulaw.com/chl/7c7e81f43957c58bbdfb.html)法律，把政府活动全面纳入法治轨道。各级政府要依法接受同级人大及其常委会的监督，自觉接受人民政协的民主监督，主动接受社会和舆论监督，让权力在阳光下运行。要坚持科学、民主、依法决策，认真听取人大代表、政协委员意见，听取民主党派、工商联、无党派人士和各人民团体意见，听取社会公众和企业意见。支持工会、共青团、妇联等群团组织更好发挥作用。全面落实行政执法责任制和问责制，对一切违法违规的行为都要坚决查处，对一切执法不公正不文明的现象都要坚决整治，对所有行政不作为的人员都要坚决追责。（国务院各部门分别负责）
　　88.全面推进政务公开。贯彻实施新修订的《[中华人民共和国政府信息公开条例](https://www.pkulaw.com/chl/117226c0d8725a33bdfb.html)》，推进基层政务公开标准化规范化建设。（国务院办公厅牵头，有关部门按职责分工负责）
　　（四十四）深入推进党风廉政建设。
　　89.扎实开展“不忘初心、牢记使命”主题教育。认真贯彻落实中央八项规定及其实施细则精神，持之以恒纠治“四风”。加强廉洁政府建设，一体推进不敢腐、不能腐、不想腐。强化审计监督。政府工作人员要自觉接受法律监督、监察监督和人民监督。（国务院各部门分别负责）
　　90.压减和规范督查检查考核事项。实施“互联网＋督查”。（国务院办公厅牵头）减少开会和发文数量，今年国务院及其部门要带头大幅精简会议、坚决把文件压减三分之一以上。（国务院各部门分别负责）
　　（四十五）切实强化责任担当。
　　91.健全激励约束机制和尽职免责机制，营造干部愿干事、敢干事、能干成事的环境。更好发挥中央和地方两个积极性，尊重基层和群众首创精神，为地方大胆探索提供激励、留足空间。（国务院各部门分别负责）

　　**十三、**做好民族、宗教、侨务、国防、港澳台、外交工作
　　（四十六）全面贯彻党的民族政策。
　　92.坚持和完善民族区域自治制度，深化民族团结进步教育，铸牢中华民族共同体意识，促进各民族和睦相处、和衷共济、和谐发展。加大对民族地区和人口较少民族发展的支持，深入实施兴边富民行动。（国家民委牵头）
　　（四十七）全面贯彻党的宗教工作基本方针。
　　93.坚持我国宗教的中国化方向，依法管理宗教事务，发挥宗教界人士和信教群众在促进经济社会发展中的积极作用。（有关部门按职责分工负责）
　　（四十八）认真落实侨务政策。
　　94.保障海外侨胞和归侨侨眷合法权益，改善和加强服务，发挥好他们的独特优势和重要作用。（有关部门按职责分工负责）
　　（四十九）扎实推进国防和军队建设。
　　95.继续以党在新时代的强军目标为引领，牢固确立习近平强军思想在国防和军队建设中的指导地位，深入推进政治建军、改革强军、科技兴军、依法治军。坚持党对军队绝对领导的根本原则和制度，全面深入贯彻军委主席负责制。贯彻新时代军事战略方针，提高实战化军事训练水平，坚决维护国家主权、安全、发展利益。继续深化国防和军队改革，建立健全中国特色社会主义军事政策制度体系。（有关部门按职责分工配合做好工作）加强和完善国防教育、国防动员体系建设，增强全民国防意识。（有关部门按职责分工负责）深入实施军民融合发展战略，加快国防科技创新步伐。（国家发展改革委、工业和信息化部、国家国防科工局等按职责分工负责）各级政府要大力关心支持国防和军队建设，深入开展“双拥”活动。（退役军人部牵头）
　　（五十）继续全面准确贯彻“一国两制”、“港人治港”、“澳人治澳”、高度自治的方针。
　　96.严格依照[宪法](https://www.pkulaw.com/chl/7c7e81f43957c58bbdfb.html)和基本法办事。全力支持香港、澳门特别行政区政府和行政长官依法施政。（国务院港澳办牵头）支持港澳抓住共建“一带一路”和粤港澳大湾区建设的重大机遇，更好发挥自身优势，全面深化与内地互利合作。（国务院港澳办、外交部、国家发展改革委、商务部等按职责分工负责）
　　（五十一）坚持对台工作大政方针。
　　97.全面贯彻落实习近平总书记在《告台湾同胞书》发表40周年纪念会上的重要讲话精神，坚持一个中国原则和“九二共识”，推动两岸关系和平发展、推进祖国和平统一进程。坚决反对和遏制“***”分裂图谋和行径，坚决维护国家主权和领土完整。深化两岸融合发展，持续扩大两岸经济文化交流合作。（有关部门按职责分工负责）
　　（五十二）坚定不移走和平发展道路、奉行互利共赢的开放战略。
　　98.坚定维护多边主义和以联合国为核心的国际体系。积极参与全球治理体系的改革完善，坚定维护开放型世界经济，推动构建人类命运共同体。加强与主要大国沟通对话与协调合作，深化同周边国家关系，拓展与发展中国家互利合作。积极为妥善应对全球性挑战和解决地区热点问题提供更多中国建设性方案。（外交部牵头）
　　今年是新中国成立70周年，是全面建成小康社会、实现第一个百年奋斗目标的关键之年。各地区、各部门要在以习近平同志为核心的党中央坚强领导下，以习近平新时代中国特色社会主义思想为指导，全面贯彻党的十九大和十九届二中、三中全会精神，树牢“四个意识”，坚定“四个自信”，坚决做到“两个维护”，自觉在思想上政治上行动上同以习近平同志为核心的党中央保持高度一致，以对人民高度负责的精神，不折不扣抓好落实，奋力完成全年经济社会发展主要目标任务，确保兑现政府对人民的承诺。一要突出重点。各部门一把手要亲自抓、负总责，对照分工逐项制定落实方案，于4月15日前报国务院。要紧扣深化改革开放、简政减税降费、优化公平营商环境、培育新动能等重点工作，尽快出台细化措施。二要抓紧推进。已确定的工作和政策要尽快落地、资金尽快下达，坚持结果导向，及时了解政策实施中的企业反应、群众呼声，确保工作早见效、市场主体有感受。三要协同发力。各部门要从大局出发，加强相互配合和政策配套，做到精准施策、务实管用。涉及跨领域、多部门参与的工作，牵头部门要发挥主导作用，主动协调推进，协办部门要积极配合，形成工作合力。四要完善督办机制。各部门要健全台账，明确相关要求，坚决防止工作推进中“跑偏走样”、不作为等问题，确保“事事有人盯、件件有着落”。同时，要加强对各地区的指导服务，减轻基层负担。各省（区、市）人民政府要主动对接国务院有关部门，围绕《政府工作报告》重点工作，结合本地区实际进一步明确重点，抓好落实。国务院办公厅要完善《政府工作报告》督查总台账，定期对账督办，结合组织开展国务院大督查、专项督查、“互联网＋督查”和专题调研，推动各项重点工作落到实处、取得实效。

国务院
2019年3月29日

© 北大法宝：（[www.pkulaw.com](https://www.pkulaw.com/)) 专业提供法律信息、法学知识和法律软件领域各类解决方案。北大法宝为您提供丰富的参考资料，正式引用法规条文时请与标准文本核对。
欢迎查看所有[产品和服务](https://www.pkulaw.com/helps/2.html)。法宝快讯：[如何快速找到您需要的检索结果？](https://www.pkulaw.com/helps/70.html)[法宝](https://www.pkulaw.com/helps/69.html)V6有何新特色？

扫描二维码阅读原文

**原文链接：**<https://www.pkulaw.com/chl/8b4f59a2d15d2fcbbdfb.html>
